# Otoscleroma of the Middle Ear and Mastoid Cavity with Facial Palsy: A Case Report

**DOI:** 10.22038/ijorl.2024.79380.3675

**Published:** 2025

**Authors:** Ahmed Galal, Ahmed Abdelbaki, Hanan Tayel, Hesham Mustafa Abdel-Fattah

**Affiliations:** 1 *Department of Otorhinolaryngology, Faculty of Medicine, Alexandria University, Egypt.*; 2 *Department of Pathology, Faculty of Medicine, Alexandria University, Egypt.*

**Keywords:** Otoscleroma, rhinoslceroma, Facial palsy, Subtotal petrosectomy, Facial nerve decompression

## Abstract

**Introduction:**

Scleroma is a chronic, specific granulomatous disease that affects the head and neck mucosa. Its common sites are the nose and larynx; however, it might affect other areas. One of the rare sites to be affected is the middle ear and mastoid cavity, for which the term otoscleroma was coined. We present such a rare case in this report.

**Case Report:**

A 47-year-old patient with a history of both old laryngoscleroma and recent Rhinoscleroma. He presented with symptoms of ear discharge and facial palsy. Examination revealed complete facial nerve palsy and an external auditory canal polyp. A decision was made to perform subtotal petrosectomy with facial nerve decompression and maximum debulking to be sent for histopathology. The result came typical of otoscleroma

**Conclusion:**

Otoscleroma is a fairly rare occurrence. It might be primary, with no evidence of Scleroma in other sites or following rhino and/or laryngoscleroma. It might be unilateral or bilateral. It might mimic the clinical picture of chronic suppurative otitis media and its complications. Tympano-mastoidectomy is recommended to stop the discharge, obtain a proper biopsy and decompress the facial nerve if needed. Otoscleroma should be suspected in the case of previous Scleroma in other sites, and otitis media or complications like manifestations.

## Introduction

Rhinoscleroma is a chronic specific granulomatous inflammation. Its causative organism is klebsiella rhinoscleromatis. It affects mainly the upper respiratory tract mucosa (1). Hence its name, it mainly affects the nose, followed by the pharynx and larynx (2,3). However, it sometimes affects unusual sites such as the lacrimal gland, trachea, sinuses and rarely the ear (4,6-8). The youngest age reported was a case of a newborn infant, and the term "scleroma neonatorum" was suggested (9). The oldest age reported was a man of 68 years (10). 

Abou-bieh et al. coined the term otoscleroma for those cases affecting the middle ear ± the mastoid cavity (11). Otoscleroma is a fairly rare disease. To our knowledge, only four reports were published, which included in total 7 cases of otoscleroma (8,11-13). 

In these reports, Otoscleroma represented only 0.25 to 1% of all Scleroma cases. Otoscleroma was either isolated primarily or associated with Scleroma of the nose and pharynx or larynx (8,11-13).

## Case Report

We report the case of a 47-year-old nonsmoker male patient. He had no past medical history. He developed a vocal cord Scleroma 15 years ago. Recently, he developed a nasal mass. An excisional biopsy was performed, and the lesion proved to be a Rhinoscleroma. As per family history, his brother also had a history of Rhinoscleroma.

He presented to our otology unit 2 months after his nasal surgery. He reported bilateral progressive hearing loss and tinnitus over the course of 2 years. Symptoms were more prominent on the left side. He also complained of left-sided otorrhea for the last couple of months prior to the presentation. It was offensive, profuse, and yellowish. At that time also, the patient started developing otalgia that was throbbing in nature. 

He additionally complained of a single attack of vertigo 1 month before the presentation. Two weeks prior to the presentation, the patient started to develop facial palsy. 

Otoscopic examination revealed the Left EAC polyp obstructing the view to any deeper structures (Fig 1A). On the other hand, the right ear revealed a dull tympanic membrane. Tympanometry revealed a bilateral low basic volume type B. Audiometry showed bilateral moderate conductive hearing loss with an average of 38.75 dB right and 42.5 dB left. 

**Fig 1:**
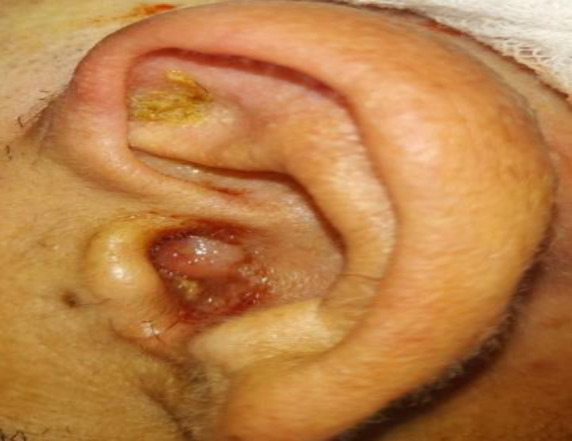
**A** **-** left EAC polyp.

High-resolution computed tomography (HRCT) revealed total opacification of the middle ear cleft and mastoid on the right side. On the left side, total mastoid and middle ear opacification, facial nerve dehiscence, ossicular erosion, tympanic membrane effacement and mastoid bone erosion were observed (Figure 1B).

**Fig 1:**
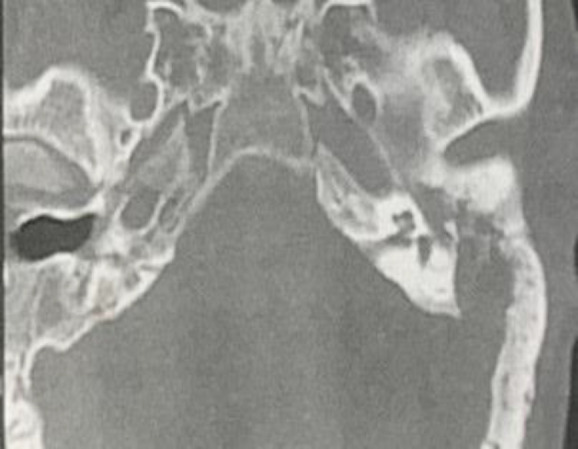
**B** **-** HRCT petrous bone, bone window, axial view showing bilateral total opacification of the middle ear cleft and mastoid on the right side. On the left side, additional ossicular erosion, tympanic membrane effacement and mastoid bone erosion were observed.

Magnetic resonance imaging (MRI) revealed a left-loculated mastoid lesion with total opacification of the mastoid and middle ear cavities (Figure 1C). In addition, signal loss in the vestibule and semicircular canals was detected. Radiologically, the preliminary diagnosis was otitis media complicated by mastoid abscess and labyrinthitis ossificans. 

**Fig 1:**
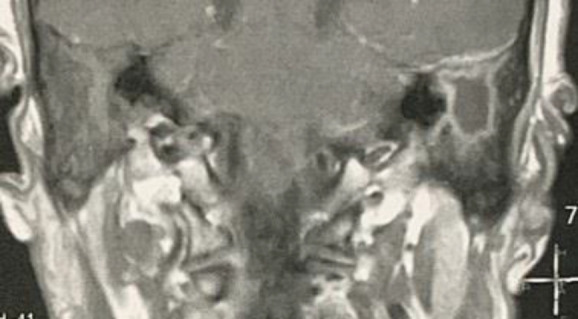
**C** **-** MRI coronal T1 post contrast showing left loculated mastoid lesion with rim enhancement and total opacification of both the mastoid and middle ear cavities.

Surgery:

A decision was taken to perform an exploratory tympano-mastoidectomy with facial nerve decompression under general anaesthesia. The goal was to debulk this soft tissue mass, define its histopathology and decompress the insulted facial nerve. Informed consent was obtained from the patient for both surgery and reporting his case.

Post-auricular approach was chosen. The polypoidal tissue was seen invading most of the skin of the external auditory canal (EAC) and the whole tympano-meatal flap (TMF) (Figure 2A). The middle ear and mastoid were also full of disease. The pathology was grossly dark red and quite vascular in nature. 

**Fig 2:**
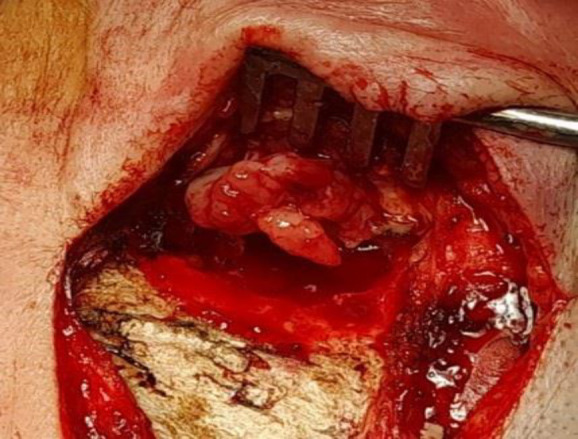
**A**- Polyp filling the whole EAC intra-operatively after post-auricular skin incision.

EAC skin and tympanic membrane were all eroded by the disease. Ossicles were eroded. Lateral and posterior semicircular canals were invaded up to the vestibule, which was also filled with disease. Following the disease, the labyrinthine bone was eroded and invaded. Consequently, hearing would be expected to be lost in this ear. Therefore, an intraoperative decision was taken to perform a subtotal petrosectomy. Facial nerve decompression was then initiated. It was found to be edematous, swollen, inflamed, and reddish, and granulation tissues were adherent to it at the labyrinthine portion and geniculate ganglion. The tympanic segment was dehiscent and, with its sheath, opened up spontaneously. The polyps were sent for histopathological examination after maximum debulking (Figure 2B). The ear was closed blind (cul de sac).

**Fig 2:**
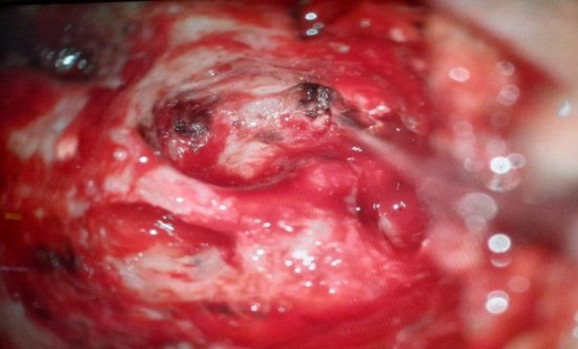
**B-** Final cavity after maximum debulking.

The patient's biopsy showed typical Scleroma histopathological features. These included chronic inflammatory cells and foamy macrophages (Mikulicz cells) in the stroma mixed with plasma cells and lymphocytes. Gram-negative bacilli were also detected in the Mikulicz cells (Figure 3 A, B, C).

**Fig 3:**
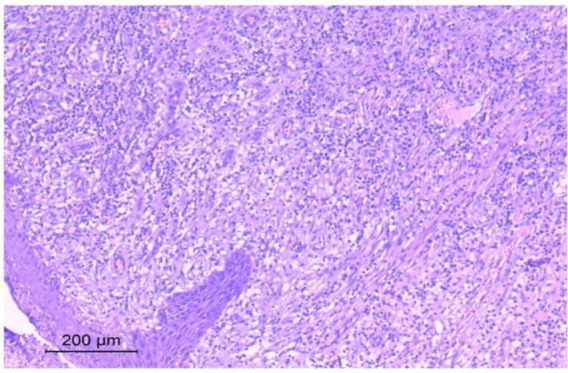
**A** **-** Tissue covered by stratified squamous epithelium with numerous chronic inflammatory cells and foamy macrophages (Mikulicz cells) in the stroma. (H&E x100)

**Fig 3:**
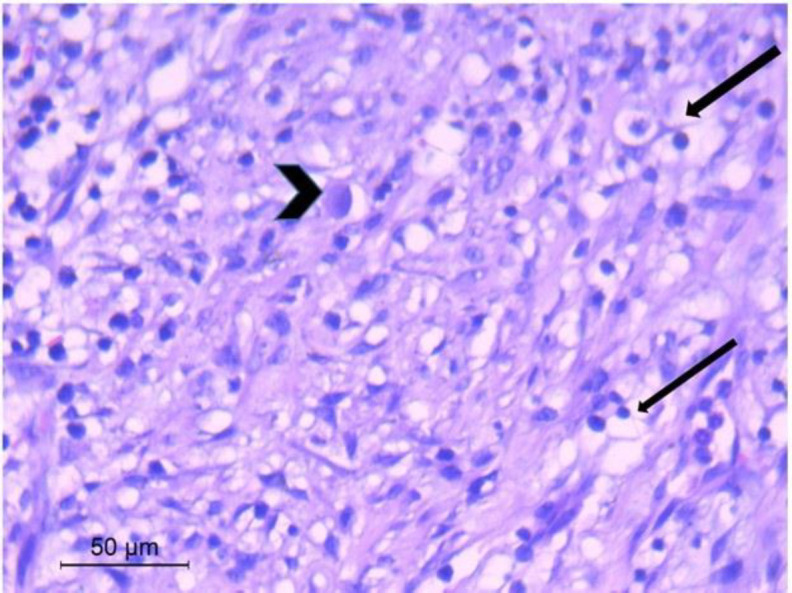
**B**- High power view demonstrating Mikulicz cells (arrow) admixed with plasma cells (head arrow) and lymphocytes. (H&Ex400)

**Fig 3:**
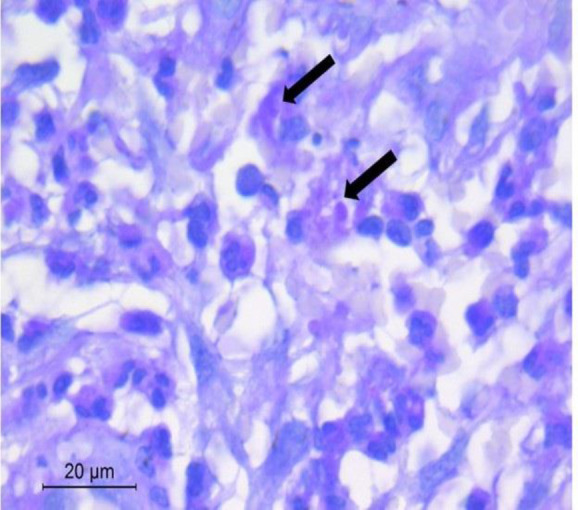
**C** **-** Another high-power view demonstrating gram-negative bacilli in Mikulicz cells arrows. (PAS x1000)

Regarding follow-up, 17 months after surgery, the patient had a few sporadic mild attacks of dizziness, the last of which occurred 3 months ago and resolved spontaneously. He has had no vestibular symptoms since then without any medical treatment. His facial nerve improved to House Brackmann grade II. 

## Discussion

According to various reports, Otoscleroma is a rare occurrence affecting less than 1% of total Scleroma cases. In our review of the English literature, only four papers reporting seven cases could be found. Reports came from only two countries: Egypt, where there were 5 cases, and India, where there were 2 cases (8,11-13).

Of these, 5 were primary, with no evidence of nasal, pharyngeal, or laryngeal disease even after blind laryngeal and nasopharyngeal biopsies (8, 11-13). One was associated with nasal and the other with nasal and pharyngeal disease (11,12). 

The sexes were fairly distributed (3 females and 4 males). Six patients were adults, ranging in age from 18 to 40 (8,11-13).

A single case was a child of 10 years who was from India (8). Our reported case was an adult male with a history of both an old (15 years) laryngeal disease and a recent nasal disease (2 months).

Five patients were reported unilateral. Only two were bilateral (11,12). In the present study, the contralateral side had a middle ear effusion, opacification on HRCT and a large ABG. Therefore, it is probable that there is a bilateral affection in our patient. Nonetheless, due to lack of polyp, perforation or discharge and the fact that the patient has contralateral facial palsy, and the ear canal is closed blind, we preferred to wait and see for this ear before venturing into surgery on a better hearing and better facial nerve side.

Our patient presented with facial palsy, which concurs with only one of the reported cases (12).

On the other hand, another author reported affection of the facial nerve bony canal intra-operatively but without clinical paralysis (13).

All reported patients presented with mucopurulent otorrhea and hearing loss, similar to our patient. However, only four of the seven reported cases presented with an aural polyp, as in our current report (8,11,13). The other three patients presented with draining ears with tympanic membrane perforations (11-13). This could be confusing with otitis media and its complications.A study suggested that Scleroma arises at a junctional epithelial line, i.e. a line where two epithelia join. Thus, Rhinoscleroma starts at the line where the stratified squamous epithelium of the vestibule joins the columnar respiratory epithelium of the nose proper, and Laryngoscleroma starts below the vocal cord where the stratified squamous epithelium of the cord merges with the columnar ciliated epithelium in the subglottic area (4). 

In the middle ear, the part of the tympanic cavity embryologically represented by the tubo-tympanic recess is lined by columnar ciliated epithelium. The rest of the middle ear cavity is lined by flattened and non-ciliated epithelium (14). This line of junction of the two epithelia may explain the origin of otoscleroma on the same basis. Management of all reported cases, similar to our approach, was surgical. This is most likely based on the fact that these patients presented with a discharging ear resistant to treatment and would need tympano-mastoid surgery for cleaning purposes and biopsy for proper diagnosis histopathologically, regardless of the diagnosis. Additionally, in the case of recent facial palsy, a facial nerve decompression would be needed. In all cases, dark red granulation tissue was found, debulked and sent for histopathological assessment, which would later reveal features of otoscleroma. This was typical of the scenario encountered with our current patient. 

## Conclusion

Otoscleroma is a rare occurrence. It presents with typical features of chronic ear and even with its complications, such as facial palsy. A high index of suspicion should arise in case of previous Scleroma in the nose and/or pharynx and larynx. However, it is highly probable that it presents alone. Surgery is essential to treat the drainage, obtain a biopsy for accurate diagnosis, and decompress the facial nerve if affected. 
